# Efficacy of brentuximab vedotin combined with sintilimab in relapsed/refractory DLBCL patient with secondary hemophagocytic syndrome: a case report and literature review

**DOI:** 10.3389/fonc.2025.1531713

**Published:** 2025-09-23

**Authors:** Siyu Cheng, Huihong Wu, Wenguang Bao, Zhigang Peng, Fengxiang Huang, Shengsheng Zhou, Qinli Mo, Ye Li, Yehong Bin, Dong Lan, Haiyan Yang

**Affiliations:** Department of Medical Oncology, The First Affiliated Hospital of Guangxi Medical University, Nanning, Guangxi, China

**Keywords:** HLH, relapsed/refractory diffuse large B-cell lymphoma, hemophagocytic syndrome, brentuximab vedotin, sintilimab

## Abstract

Currently, there is no definitive and effective treatment strategy for relapsed/refractory diffuse large B-cell lymphoma (R/R DLBCL). In recent years, studies on brentuximab vedotin (BV) and programmed cell death-1 (PD-1) monotherapy for R/R DLBCL have demonstrated significant clinical benefits. Based on this, this article retrospectively analyzes a case of R/R DLBCL with secondary hemophagocytic syndrome successfully treated with BV combined with a PD-1 monoclonal antibody and reviews the relevant literature. The patient was a 55-year-old woman who was diagnosed with stage IIE diffuse large B-cell lymphoma in June 2020. She failed to achieve complete remission during first-line treatment with the R-CHOP (rituximab, cyclophosphamide, doxorubicin/epirubicin, vincristine, and prednisone) regimen. After switching to the R2-GDP regimen for second-line salvage therapy, her condition continued to progress, and recurrent hemophagocytic syndrome developed. Subsequent treatment with BV combined with a PD-1 monoclonal antibody resulted in significant relief of her symptoms. As of the follow-up on 8 March 2025, the patient maintained a normal life and had no intolerable immune-related adverse effects. This study suggests that BV combined with PD-1 monoclonal antibody may exert a synergistic effect in the treatment of R/R DLBCL complicated with hemophagocytic lymphohistiocytosis (HLH).

## Introduction

Diffuse large B-cell lymphoma (DLBCL) is the most common subtype of non-Hodgkin lymphoma (NHL), accounting for approximately 33% of NHL cases in developed countries (1). Although first-line R-CHOP (rituximab, cyclophosphamide, doxorubicin/epirubicin, vincristine, and prednisone) therapy can cure the majority of patients with DLBCL, approximately 40% eventually experience relapse or refractoriness, progressing to relapsed/refractory DLBCL (R/R DLBCL) (2, 3). Notably, patients with R/R DLBCL are highly prone to developing hemophagocytic lymphohistiocytosis (HLH) as a complication, driven by uncontrolled inflammatory responses triggered by malignant cells. Once HLH develops, conventional HLH treatment regimens often yield limited efficacy. Given these limitations of standard therapies, exploring novel therapeutic approaches with distinct mechanisms of action is imperative. In this context, monoclonal antibodies targeting the programmed cell death-1 (PD-1)/programmed death ligand-1 (PD-L1) pathway offer a promising strategy: by reversing tumor-mediated immune suppression, they reactivate anti-tumor immune responses. Such agents have shown considerable therapeutic value in the management of R/R DLBCL complicated by HLH.

PD-1 is a receptor protein expressed on immune T cells, which primarily inhibits immune system activation through binding to PD-L1. PD-L1 is expressed in 11%–30% of DLBCL cells (4–7). The response rate to PD-1 monotherapy in DLBCL is only 10% (8), which may be associated with the low PD-L1 expression levels in DLBCL cells (9). Despite the poor prognosis of patients with DLBCL receiving anti-PD-1 therapy, the efficacy of PD-1 monoclonal antibodies in combination with other agents for treating DLBCL warrants further investigation.

With deeper insights into the disease and advances in science and technology, the treatment of DLBCL has entered the era of precision targeting. Numerous studies have indicated that while high CD30 expression is a hallmark of classical Hodgkin lymphoma (cHL) and anaplastic large cell lymphoma (ALCL), its expression in DLBCL is relatively low and heterogeneous, with a positive rate typically ranging from 20% to 30% (10). Despite the overall low CD30 expression in DLBCL, its tendency to be upregulated in relapsed/refractory populations—coupled with the availability of brentuximab vedotin (BV), a highly effective, mechanistically distinct targeted agent that has demonstrated significant clinical activity in pivotal trials (e.g., Study SG035-0004)—renders CD30 an important therapeutic target for R/R DLBCL (11–16). As an antibody–drug conjugate (ADC) targeting CD30, BV was officially approved by China’s National Medical Products Administration (NMPA) in 2020 for the treatment of CD30-positive relapsed/refractory cHL and ALCL, thereby providing a novel therapeutic option for CD30-positive lymphomas (17, 18).

In this article, we retrospectively analyzed the clinical features, treatment course, and disease regression of a patient with R/R DLBCL complicated by HLH who received BV in combination with a PD-1 monoclonal antibody. Additionally, we reviewed relevant literature to explore the efficacy and safety of this combined regimen in the management of R/R DLBCL with concurrent HLH.

## Case report

A 55-year-old female patient was admitted to the hospital on 15 June 2020, with a 5-month history of right lower quadrant abdominal pain and a 20-day history of diarrhea. She first developed recurrent dull pain in the right lower abdomen in January 2020, which was not taken seriously at the time. Starting from 18 May 2020, the pain in the right lower quadrant became frequent and evolved into a tearing sensation, accompanied by yellowish diarrhea (approximately two to three episodes per day) and significant weight loss (B symptom). She denied experiencing fever, night sweats, or other discomfort. There were no notable findings in her past medical history, personal history, family history, or marital and reproductive history. Physical examination revealed no abnormalities.

**Figure 1:**
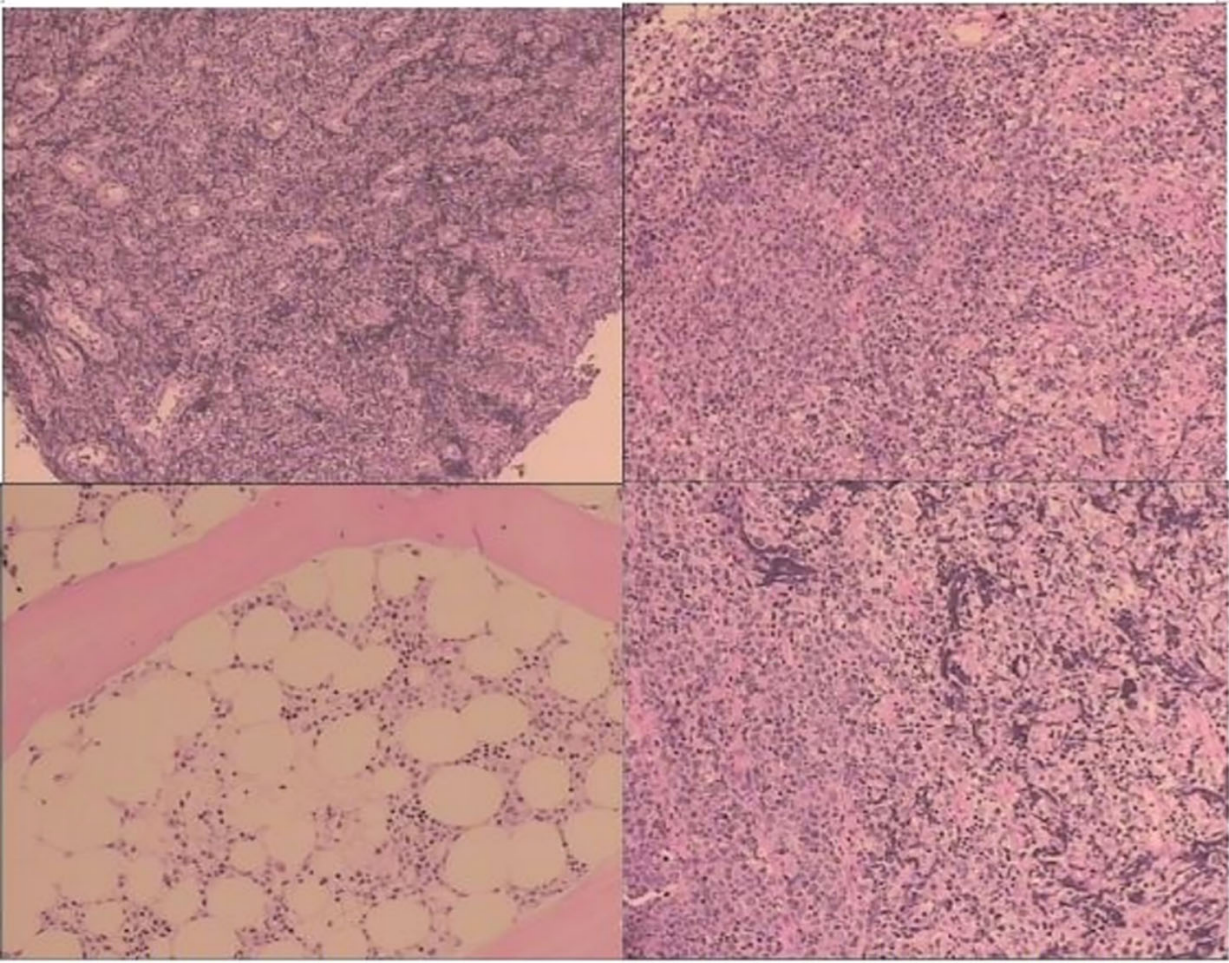
Pathological examination (mass in the terminal ileum and the ileocecal region): the positive expression of CD20*, CD79a, CD30, MUM-1, Bcl-2, Bc1-6, P53, Ki-67#, and c-Myc was identified using immunohistochemistry; CD10, CD21/CD23, CD3, CD5, CyclinD1, and CK all express negatively using immunohistochemistry.

Comprehensive relevant examinations were performed during hospitalization:

1. Pathological examination (**Figure 1**): The results confirmed non-germinal center B-cell-like (non-GCB) DLBCL, with sparse CD30 expression. Genetic testing revealed mutations in TP53 (51.80%), IRF4 (19.29%), BIG1 (35.18%), TMSB4X (8.33%), IGLL5 (51.80%), and PCL0 (17.07%).
2. Laboratory tests: Red blood cell (RBC) count, 3.24×10¹²/L; hemoglobin (Hb) count, 88.50 g/L; neutrophil (Neu), count 1.08×10^9^/L; lactate dehydrogenase (LDH), 205 U/L. Fecal occult blood test was positive.
3. Bone marrow aspiration and biopsy: No evidence of lymphoma infiltration.
4. PET/CT: (i) Multiple lymphomatous lesions involving the cecum, ascending colon, colon, and right para-aortic region [maximum standardized uptake value (SUVmax) 7.5]; (ii) mild lymph node hyperplasia in both inguinal regions.

Based on the 2016 WHO Classification of Lymphoid Neoplasms, the patient was diagnosed with DLBCL. According to the Hans algorithm, she was classified as having the non-germinal center B-cell-like subtype. Per the Ann Arbor staging system, her disease was staged as IIE, with B symptoms; her International Prognostic Index (IPI) score was 1.

In summary, the patient was categorized into the low-intermediate-risk group. She was suspected to have double-expressor DLBCL and met the criteria for first-line R-CHOP therapy. Therefore, she initiated a six-cycle first-line R-CHOP regimen on 19 June 2020 (rituximab 600 mg on day 0; cyclophosphamide 1.2 g, vincristine 2 mg, and pirarubicin 80 mg on day 1; dexamethasone 15 mg on days 1–5). During treatment, she developed grade II neutropenia after the first cycle of chemotherapy.

To assess treatment response, a PET/CT scan was performed on 23 December 2020, which showed residual tumor tissue in the ileocecal and ascending colon regions, though with a reduced area compared to prior imaging. Since complete remission (CR) was not achieved, a six-cycle second-line R2-GDP regimen was initiated on 24 December 2020 (rituximab 600 mg on day 1; gemcitabine 1.6 g on days 1 and 7; cisplatin 60 mg on days 1 and 2; dexamethasone 40 mg on days 1–4; lenalidomide 25 mg on days 1–10).

Post-treatment follow-up PET/CT revealed that most lesions had improved, with a significant reduction in SUV values of a 1.6-cm lymph node adjacent to the ascending colon compared to the original ileocecal lesion. Overall, the patient achieved a partial remission (PR). It was recommended that she receive local radiotherapy targeting the residual small lesions near the ascending colon after completing six cycles of the second-line chemotherapy.

**Figure 2:**
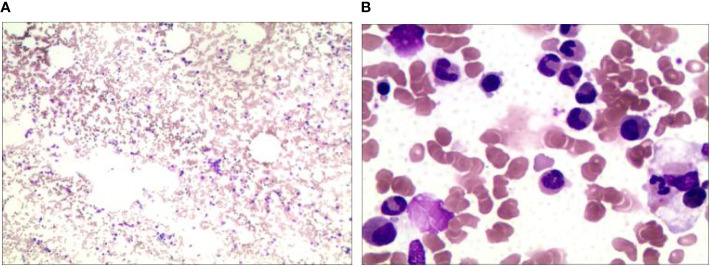
Bone marrow cytomorphologic findings in a patient with relapsed/refractory diffuse large B-cell lymphoma with hemophagocytic syndrome. Rhett’s staining **(A)** ×20, **(B)** ×100. Bone marrow hyperplasia is active and hemophagocytic cells are seen on the whole film.

**Figure 3:**
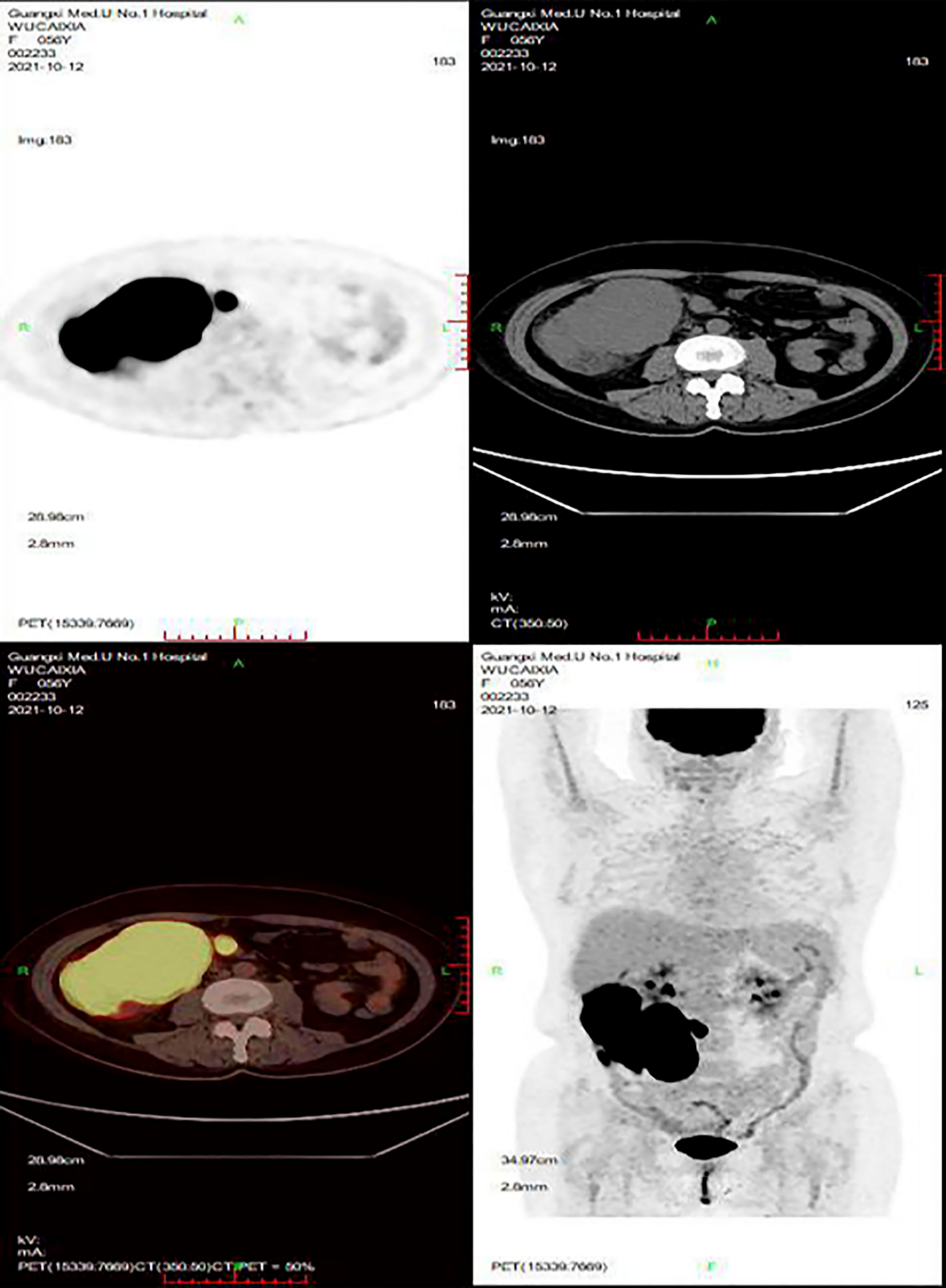
PET/CT. After lymphoma chemotherapy: **(A)** The lesions in the ileocecal region, the colon, and the mesentery were more advanced than before. **(B)** The newly developed right anterior lymph node lesions of the diaphragm.

In June 2021, the patient presented with fever and a palpable right abdominal mass, prompting readmission for further evaluation. Relevant examinations revealed the following: (1) Laboratory tests: White blood cell (WBC) count, 3.12×10^9^/L; RBC count, 2.05×10¹²/L; platelet (PLT) count, 58.20×10^9^/L; LDH, 382 U/L; CA125, 47.20 U/mL; serum ferritin, 3,104.9 ng/mL; and triglycerides, 3.09 mmol/L. (2) Bone marrow aspiration: Active bone marrow hyperplasia with hemophagocytic cells was observed throughout the smear (**Figures 2A, B**). (3) PET/CT (**Figure 3**): Post-chemotherapy findings for lymphoma included the following: (i) Progression of lesions in the ileocecal region, colon, and mesentery compared to prior scans; and (ii) newly emerging right anterior diaphragmatic lymph node lesions. Given the patient’s failure to achieve CR with prior first-line and second-line therapies, coupled with progressive abdominal lesions, she was diagnosed with R/R DLBCL complicated by hemophagocytic syndrome.

**Figure 4:**
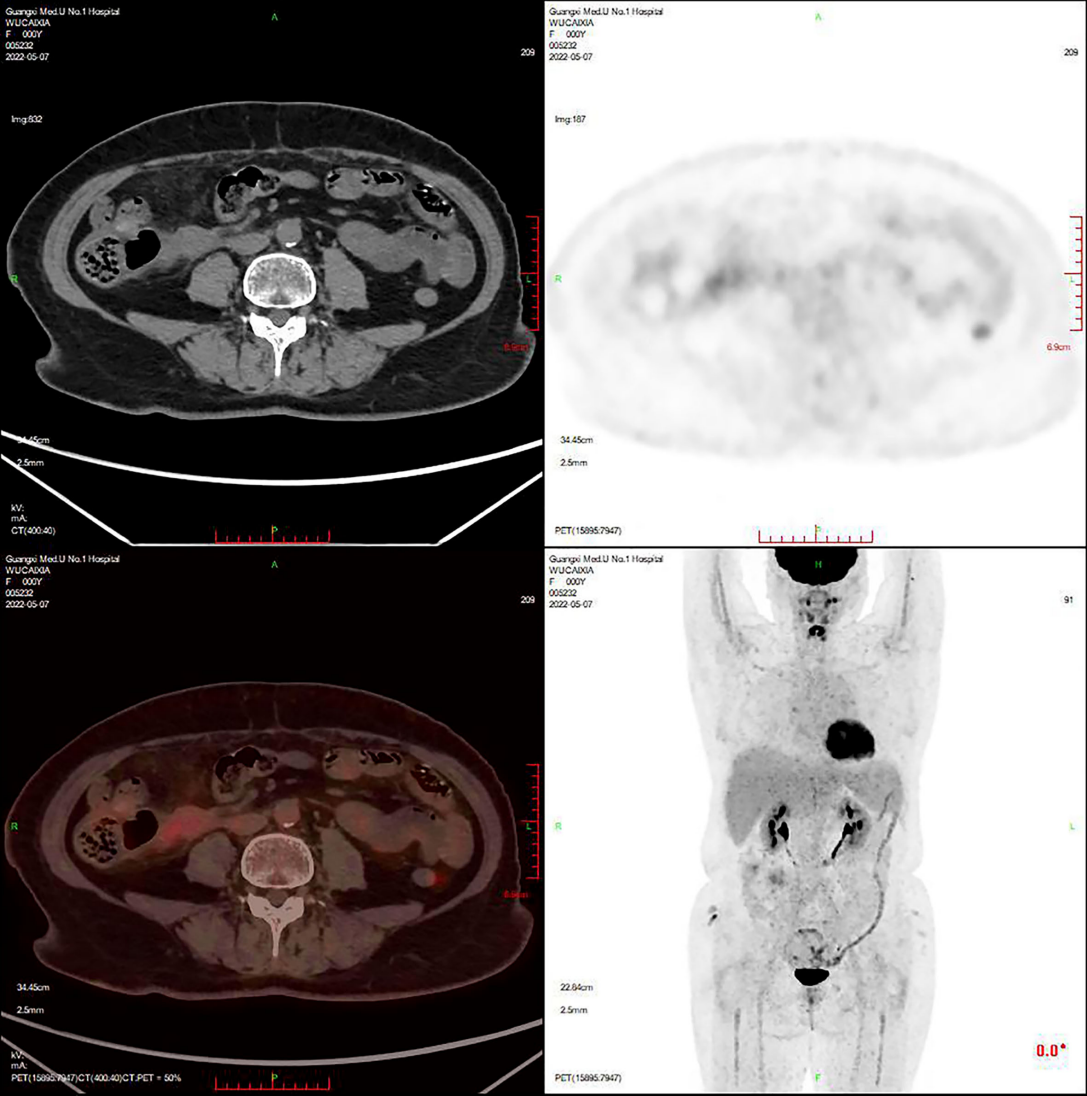
PET/CT. After chemotherapy and targeted treatment of lymphoma: **(A)** The right anterior lymph node lesions of the protodiaphragm have been absorbed and inactivated. **(B)** The primary ileocecal, colon, and mesenteric lesions were obviously improved; most of them were absorbed and inactivated, but tumor active tissues were still found in the ileocecal region. **(C)** No definite new lesions were found (Deauville score: 4 points).

On 5 November 2021, the patient initiated the first cycle of chemotherapy comprising liposomal doxorubicin (60 mg on day 1), zanubrutinib (160 mg twice daily orally on days 1–14), paclitaxel (0.4 g on day 5), decitabine (10 mg on days 1–5), and lenalidomide. Following chemotherapy, she developed grade IV myelosuppression, accompanied by multiple perineal and sacrococcygeal skin ulcerations with localized purulence. On 27 December 2021, the patient presented with recurrent fever; her condition was assessed as progressive lymphoma and recurrent hemophagocytic syndrome. On 3 January 2022, she commenced third-line DEP chemotherapy to control the hemophagocytic syndrome. However, the patient continued to experience recurrent fevers, and hemophagocytic markers failed to normalize. Given the previously detected positive CD30 expression, she was deemed eligible for BV-targeted therapy. On 19 January 2022, the patient initiated a regimen of BV combined with a PD-1 monoclonal antibody (BV 200 mg on day 1 + PD-1 monoclonal antibody 200 mg on day 1, every 3 weeks) for five cycles. Post-treatment, her symptoms improved significantly. A repeat PET/CT on 7 May 2022 (**Figure 4**) confirmed a PR. Follow-up was conducted until 8 March 2025, during which no recurrence of HLH or disease progression was observed. The patient attended regular hospital visits to receive maintenance therapy with the BV plus PD-1 regimen. By the final follow-up, she had resumed a normal life with no grade 3 or higher immune-related adverse events (**Table 1**).

**Table 1:** Therapeutic timeline of the patient with R/R DLBCL and secondary hemophagocytic syndrome.

Time period	Treatment phase	Regimen	Outcome
Jun 2020	Diagnosis	- PET/CT: Multifocal lymph node involvement (ileocecal, colon, mesentery)	Ann Arbor stage IIE, IPI score 1 (low-intermediate risk)
		- Pathology: Non-GCB DLBCL, focal CD30 expression	
Jun–Dec 2020	First-line therapy	R-CHOP ×6 cycles:	Partial remission (PR) on PET/CT (Dec 2020)
		- Rituximab 600 mg (day 0), cyclophosphamide 1.2 g (day 1), epirubicin 80 mg (day 1), vincristine 2 mg (day 1), prednisone 15 mg (days 1–5)	
Jan–Jun 2021	Second-line therapy	R2-GDP ×6 cycles:	Stable disease; PR maintained
		- Rituximab 600 mg (day 1), gemcitabine 1.6 g (day 1/7), cisplatin 60 mg (days 1 and 2), dexamethasone 40 mg (days 1–4), lenalidomide 25 mg (days 1–10)	
Jun 2021	Disease progression	- New abdominal mass detected	Diagnosis of relapsed/refractory DLBCL with secondary hemophagocytic syndrome
		- PET/CT: Progressive ileocecal lesions and new diaphragmatic lymphadenopathy	
		- Bone marrow biopsy: Hemophagocytosis	
Nov 2021–Jan 2022	Third-line therapy	Liposomal doxorubicin (60 mg day 1) + zanubrutinib (160 mg bid days 1–14) + paclitaxel (0.4 g day 5) + decitabine (10 mg days 1–5)	Treatment failure (persistent fever, disease progression)
Jan 2022	Salvage therapy	- Initial: DEP regimen (dexamethasone + etoposide + cisplatin)	DEP is invalid
		- Final: Brentuximab vedotin (200 mg day 1) + PD-1 inhibitor (200 mg day 1), every 3 weeks × 5 cycles	BV + PD-1: PR on PET/CT (Sep 2022; Deauville score 4)
8 March 2025	Follow-up	Continued BV + PD-1 maintenance therapy	Alive, asymptomatic (no fever, abdominal pain, or melena)

PD-1, programmed cell death-1; R/R DLBCL, relapsed/refractory diffuse large B-cell lymphoma; BV, brentuximab vedotin; NHL, non-Hodgkin’s lymphoma; CHL, classical Hodgkin lymphoma; ALCL, anaplastic large cell lymphoma; PD-L1, programmed death ligand-1.

## Discussion

R/R DLBCL shows low sensitivity to conventional chemotherapy and radiotherapy. Even with salvage chemotherapy ± radiotherapy and autologous stem cell transplantation (ASCT), the long-term relapse rate remains as high as approximately 80%. Although CD19-targeted chimeric antigen receptor T-cell (CAR-T) therapies and bispecific antibodies have significantly improved survival outcomes, their exorbitant treatment costs far exceed the financial means of average patients, resulting in severely limited global accessibility. Thus, exploring novel agents and therapeutic strategies is particularly urgent and crucial.

Both the tumor microenvironment and cancerous tissues in DLBCL are highly complex, with both expressing PD-L1 (19)—a factor associated with treatment responses and prognosis in R/R DLBCL (9). Currently, monoclonal antibodies to the PD-1/PD-L1 pathway for the treatment of R/R DLBCL are still in early-stage clinical studies, and their safety and efficacy need to be validated by more studies and longer follow-ups. The ORR of R/R DLBCL was 25% by pembrolizumab and CDK9 regimen (20). Another study observed that 27 of 30 patients with DLBCL obtained remission by pembrolizumab and R-CHOP therapy (21). The 2-year progression-free survival (PFS) was 83%, and the median follow-up duration was 25.5 months (21). This study strongly demonstrated that pembrolizumab combined with R-CHOP therapy significantly improved the CR rate and 2-year PFS in DLBCL, and the toxicity of the combination therapy was the same as that of the R-CHOP regimen. These findings establish an important foundation for future research on PD-1-based immunotherapy combined with other therapeutic agents.

It is well known that BV can significantly improve the prognosis of patients with CD30-positive peripheral T-cell lymphoma, cHL, and ALCL. Therefore, BV has been clinically approved for the treatment of these tumors. A meta-analysis on the efficacy of BV in the treatment of R/R cHL found that BV was significantly effective in the treatment of R/R cHL by reviewing 32 studies (ORR: 62.2%, 95% CI: 56.0–68.9, *I* ^2^ = 9.7%; 5-year PFS: 31.9%–33%) (22). Other studies have attained similar consequences (23, 24). In recent years, clinical studies on BV treatment of R/R DLBCL are gaining popularity, and more attempts have been made in creating new combination therapy programs, expanding indications, and exploring adverse reactions. A clinical research demonstrated that BV was strongly responsive to R/R DLBCL (OR: 44%), which was not particularly correlated with CD30 expression in tumor cells (17). In addition, this study further revealed that BV can significantly improve the prognosis of patients with R/R DLBCL, including 17% of patients (8 cases) having a CR and 27% of patients (13 cases) having a PR, with a median duration of 16.6 months (25). Later, further studies found that BV combined with other treatment regimens can significantly improve the prognosis of patients with R/R DLBCL compared with BV alone. A phase I/II multicenter trial found that patients with R/R DLBCL were significantly improved by BV combined with R-CHOP chemotherapy. Furthermore, another phase I clinical study found that BV combined with lenalidomide also improved the prognosis of patients with R/R DLBCL, with a complete response rate of 35% (95% CI, 20.7–52.6) and an overall survival of 14.3 months (95% CI, 10.2–35.6) (26).

In 2021, the first report on the combination of BV and PD-1 monoclonal antibody for relapsed/refractory Hodgkin lymphoma (R/R HL) demonstrated superior efficacy, with a PFS rate of 77%, an ORR of 85%, and a CR rate of 67%. Notably, no grade 3 or higher immune-related adverse events were observed (27). Other studies also further confirmed this conclusion (28, 29). In addition, based on its unique high CD30 expression (>80%) and the aberrant molecular features of the PD-L1 pathway, relapsed/refractory primary mediastinal large B-cell lymphoma (R/R PMBL) was shown to be treated with vibutuximab in combination with navulizumab to achieve an ORR of 73% and a CR of 70% in a controlled safety profile (≥grade 3 in the phase II CheckMate-436 trial treatment-related adverse event rate <30%) and significantly better efficacy than historical controls (30). In view of the previous studies on BV and PD-1 monoclonal antibody alone in the treatment of R/R DLBCL, they all showed controllable toxicity and significant clinical benefits. Reviewing this case, the patient was resistant to the first-line and second-line chemotherapy schemes and lenalidomide, and developed hemophagocytic syndrome. Considering that the efficacy of BV alone may be poor, we had to boldly try to treat the patient with BV combined with PD-1 monoclonal antibody. Surprisingly, the patient gained a better prognosis. Recently, we found a similar conclusion in another patient with DLBCL (31). Therefore, we speculate that BV and PD-1 monoclonal antibody may have a synergistic effect on patients with R/R DLBCL, and they show promise as first-line or second-line treatment for patients with R/R DLBCL. At the same time, we found that the patient’s CD30 expression was low. Therefore, we speculate that the therapeutic efficacy of BV may not necessarily be related to tumor CD30 expression, but this speculation needs to be confirmed by further studies.

## Conclusion

R/R DLBCL with secondary hemophagocytic syndrome (R/R DLBCL-sHLH) has an extremely poor prognosis. This case suggests that BV in combination with a PD-1 inhibitor may be a therapeutic option; however, a high degree of caution is required—immune checkpoint inhibitors by themselves may induce sHLH through hyperactivation of the T cell–macrophage axis (incidence, 0.1%–0.7%; lethality, >40%). This regimen should only be considered when ICI-related HLH is strictly excluded (serum IFN-γ >500 pg/mL or sCD25 >10,000 U/mL) and there is clear evidence of lymphoma-driven sHLH, and its risk–benefit ratio still needs to be validated in future studies.

## Data Availability

The raw data supporting the conclusions of this article will be made available by the authors, without undue reservation.
